# Blood pressure lowering enhances cerebrospinal fluid efflux to the systemic circulation primarily via the lymphatic vasculature

**DOI:** 10.1186/s12987-024-00509-9

**Published:** 2024-01-26

**Authors:** Jari Jukkola, Mika Kaakinen, Abhishek Singh, Sadegh Moradi, Hany Ferdinando, Teemu Myllylä, Vesa Kiviniemi, Lauri Eklund

**Affiliations:** 1 Oulu Center for Cell-Matrix Research, Faculty of Biochemistry and Molecular Medicine, Biocenter Oulu, University of Oulu, Oulu, Finland; 2 Opto-Electronics and Measurement Technique Research Unit, Infotech Oulu, University of Oulu, Oulu, Finland; 3 Research Unit of Health Science and Technology, University of Oulu, Oulu, Finland; 4 Oulu Functional NeuroImaging (OFNI), Diagnostic Imaging, Medical Research Center (MRC), Oulu University Hospital, Oulu, Finland; 5 Research Unit of Health Sciences and Technology (HST), Faculty of Medicine, Biocenter Oulu, University of Oulu, Oulu, Finland

**Keywords:** Cerebrospinal fluid, Blood pressure, Intracranial pressure, Meningeal lymphatics, Dorsal dura, Deep cervical lymph node ligation

## Abstract

**Background:**

Inside the incompressible cranium, the volume of cerebrospinal fluid is directly linked to blood volume: a change in either will induce a compensatory change in the other. Vasodilatory lowering of blood pressure has been shown to result in an increase of intracranial pressure, which, in normal circumstances should return to equilibrium by increased fluid efflux. In this study, we investigated the effect of blood pressure lowering on fluorescent cerebrospinal fluid tracer absorption into the systemic blood circulation.

**Methods:**

Blood pressure lowering was performed by an i.v. administration of nitric oxide donor (sodium nitroprusside, 5 µg kg^−1^ min^−1^) or the Ca^2+^-channel blocker (nicardipine hydrochloride, 0.5 µg kg^−1^ min^−1^) for 10, and 15 to 40 min, respectively. The effect of ﻿blood pressure lowering on cerebrospinal fluid clearance was investigated by measuring the efflux of fluorescent tracers (40 kDa FITC-dextran, 45 kDa Texas Red-conjugated ovalbumin) into blood and deep cervical lymph nodes. The effect of nicardipine on cerebral hemodynamics was investigated by near-infrared spectroscopy. The distribution of cerebrospinal fluid tracers (40 kDa horse radish peroxidase,160 kDa nanogold-conjugated IgG) in exit pathways was also analyzed at an ultrastructural level using electron microscopy.

**Results:**

Nicardipine and sodium nitroprusside reduced blood pressure by 32.0 ± 19.6% and 24.0 ± 13.3%, while temporarily elevating intracranial pressure by 14.0 ± 7.0% and 18.2 ± 15.0%, respectively. Blood pressure lowering significantly increased tracer accumulation into dorsal dura, deep cervical lymph nodes and systemic circulation, but reduced perivascular inflow along penetrating arteries in the brain. The enhanced tracer efflux by blood pressure lowering into the systemic circulation was markedly reduced (− 66.7%) by ligation of lymphatic vessels draining into deep cervical lymph nodes.

**Conclusions:**

This is the first study showing that cerebrospinal fluid clearance can be improved with acute hypotensive treatment and that the effect of the treatment is reduced by ligation of a lymphatic drainage pathway. Enhanced cerebrospinal fluid clearance by blood pressure lowering may have therapeutic potential in diseases with dysregulated cerebrospinal fluid  flow.

**Supplementary Information:**

The online version contains supplementary material available at 10.1186/s12987-024-00509-9.

## Background

Cerebrospinal fluid (CSF) serves as a protective fluid for the brain and spinal cord, maintains metabolic homeostasis and functions as a waste sink for the central nervous system (CNS) derived metabolic waste products [1–3]. CSF is not only confined to the superficial parts of the CNS, but it also extends deeper into brain parenchyma along the perivascular spaces of penetrating arteries, mixes with brain interstitial fluid and thus may participate in the clearance of brain metabolic waste products in the absence of parenchymal lymphatic vessels [4, 5]. From the CSF, macromolecules can be directly transferred to the bloodstream via venous sinuses [6–9] or absorbed into dural tissue and meningeal lymphatic vessels distributed along venous sinuses in the dorsal and basal skull [10–14]. Other exit pathways include the nasal lymphatic route and drainage along cranial nerves [15–21]. Many recent findings imply the essential role of the meningeal and nasal lymphatic systems [17–20, 22–25], e.g., enhancement of meningeal lymphatic function with vascular endothelial growth factor C treatment was recently demonstrated to improve cognitive behavior and facilitate the drainage of CSF into deep cervical lymph nodes (dcLN) in aged mice [26, 27].

While the relative contribution of the proposed clearance pathways in CSF clearance and underlying mechanistic principles still await further detailing, CSF production is decreased during aging and disrupted CSF flow is associated with cognitive decline in Alzheimer’s disease (AD) [28–33]. In addition, the presence of biomarkers of AD in CSF samples has relevance in disease progression predictions [34, 35]. Altogether, these findings indicate the importance of adequate CSF production, circulation, and absorption in brain homeostasis, whereas aging and diminished CSF drainage predispose accumulation of harmful metabolites due to attenuated CSF dynamics. Furthermore, increased blood pressure (BP) reduces CSF convection [36] and conversely tight control of BP can reduce the risk of mild cognitive impairment [37]. Clearly, enhancement of CSF circulation and clearance deserves further investigation.

In this study, we reasoned that modulation of brain pressure–volume balance by lowering systemic BP alters CSF absorption dynamics according to the Monro-Kellie doctrine, which describes the pressure–volume relationship of brain components inside a rigid skull [38, 39]. We studied the drainage efficiency of 40 kDa FITC-dextran (FD40) and 45 kDa Texas Red-conjugated ovalbumin injected into the cisterna magna (CM) with acute reduction of BP by intravenous (i.v.) infusions of vasodilators. We observed that acute blood pressure lowering (BPL) improved CSF absorption into the systemic circulation and that absorption was markedly reduced by ligation of an intact lymphatic drainage pathway.

## Results

### Blood pressure lowering temporarily increases intracranial pressure and enhances tracer efflux into blood

**Fig. 1:**
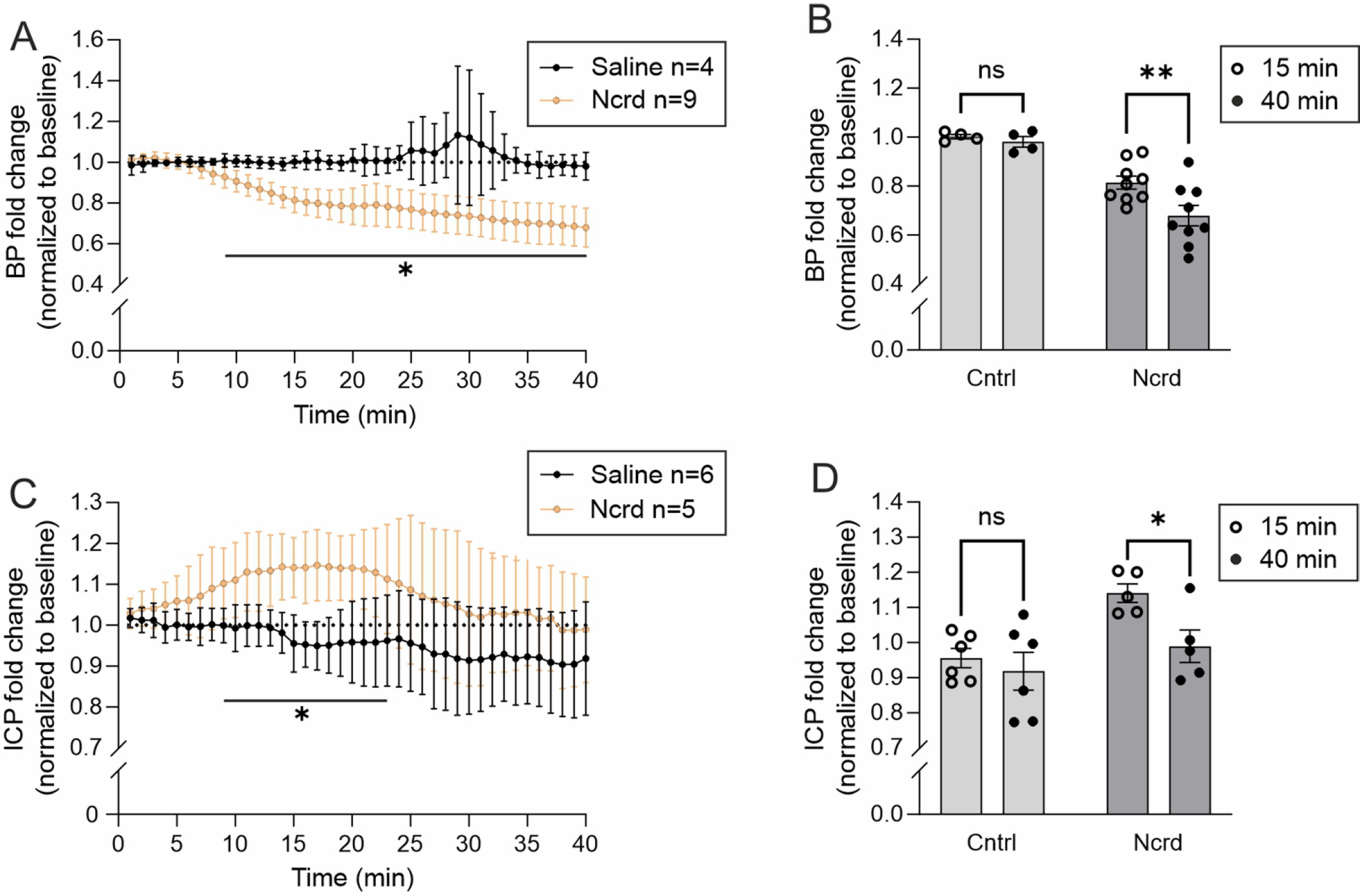
Cerebral blood and intracranial pressures during BPL. **A**, **C**, Blood pressure (BP) and intracranial pressure (ICP) during BPL with nicardipine (Ncrd) on separate groups of mice. Multiple unpaired t-tests with Welch’s corrections. **B**, **D**, Comparisons of selected timepoints in control and BPL groups. Ordinary two-way ANOVA with Šidák’s multiple comparisons. **A**–**D**, *P < 0.05, ns = non-significant. **A**, **B**, n = 4–9 mice per group. **C**, **D**, n = 5–6 mice per group. Filled and open circles in **B** and **D** represent individual mice at different timepoints taken from the graphs **A** and **C**, respectively

An i.v. injection of nicardipine resulted in a continuous decline in BP (Fig. 1A, Additional file 1: Fig. S1) starting 8 min of infusion and reaching the lowest value at 40 min (68.3 ± 19.6% of the baseline). In ~ 15 min, BP dropped by 19.0 ± 11.5% while intracranial pressure (ICP) increased significantly (+ 14.0 ± 7.0%) and returned to baseline levels within ~ 40 min (Fig. 1C). Control mice infused with saline did not show significant changes in BP or ICP (Fig. 1).

**Fig. 2:**
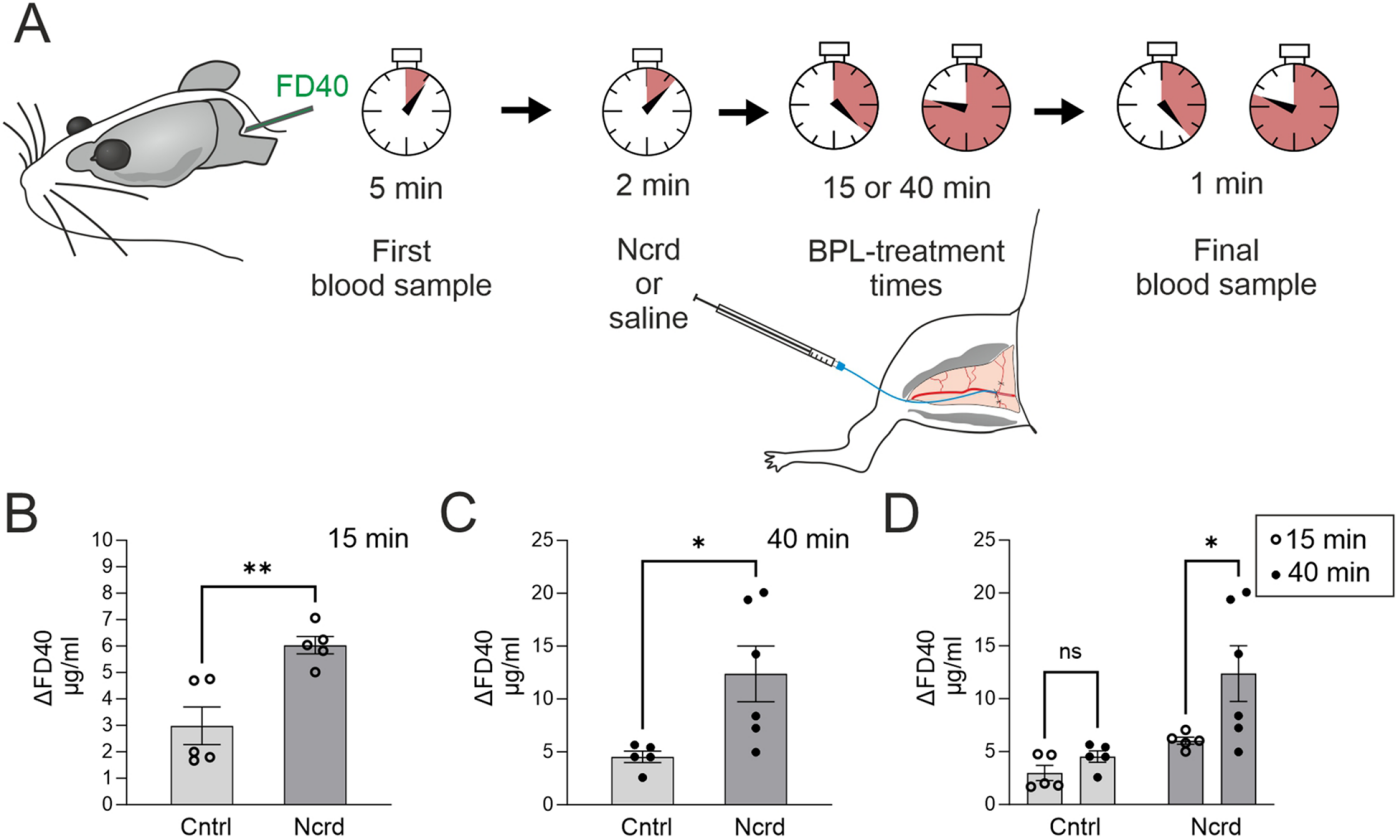
Manipulation of CSF tracer efflux with BPL. **A**, Experimental scheme and timeline to investigate the effect of BPL on CSF tracer efflux. 40 kDa FITC-dextran (FD40) injection into the cisterna magna (CM) CM was followed by blood sampling from femoral vein 5 min later, with an i.v. (femoral vein) infusion of nicardipine (Ncrd) or saline (Cntrl) starting 2 min after the first blood sampling for either 15 or 40 min, after which the final blood sampling was performed. **B**, **C**, FD40 concentration change in blood during 15-min and 40-min BPL, Mann–Whitney *U* test and unpaired t-test with Welch’s correction, respectively.** D**, Comparison of selected timepoints in control and BPL groups. Two-way ANOVA with Šidák’s multiple comparisons. **B**–**D**, *P < 0.05, ns = non-significant. n = 5–6 mice per group. Filled and open circles in **B**-**D** represent individual mice

To investigate if the increased ICP improves the drainage of macromolecules into the systemic blood circulation, we injected 4 µl of FD40 into the CM, followed by blood sampling before and at the end of BPL treatment or saline infusion (Fig. 2A). FD40 concentration increased 2.0-fold (3.0 ± 1.5 µg ml^−1^ vs 6.0 ± 1.0 µg ml^−1^) after the 15-min BPL treatment (Fig. 2B) and a 2.7-fold (4.5 ± 1.5 µg ml^−1^ vs 12.4 ± 7.5 µg ml^−1^) after the 40-min BPL treatment (Fig. 2C). No significant change in FD40 concentration was found in controls receiving only saline (Fig. 2D).

**Fig. 3:**
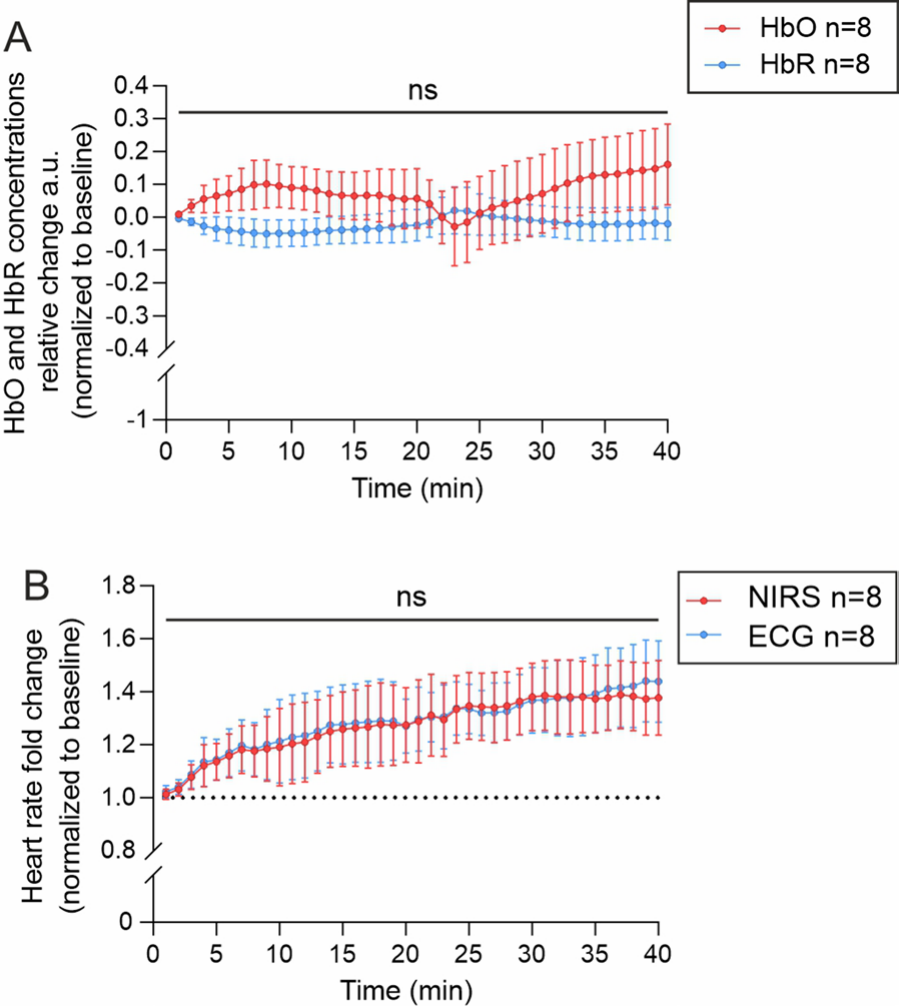
Cerebral hemodynamics during BPL. **A**, Near-infrared spectroscopy (NIRS) measurements of relative oxygenated hemoglobin (HbO) and deoxygenated hemoglobin (HbR) concentration changes during BPL, one-way ANOVA with repeated measurements and Dunnett’s multiple comparisons. **B**, NIRS signal fluctuation frequency in 785 nm wavelength corresponds with heart rate measure from electrocardiography (ECG). Multiple unpaired t-tests with Welch’s correction. **A** and **B**, ns = non-significant, the measurements are from the same mice, n = 8 mice per group

We then asked whether acute BPL reduces cerebral oxygen delivery and measured relative changes in cerebral blood oxygenation by transcranial near-infrared spectroscopy (NIRS). BPL did not affect blood oxygenation as there were virtually no changes in oxygenized and deoxygenized hemoglobin (HbO and HbR, respectively) concentrations (Fig. 3A). Heart rate estimated from NIRS signals were in good agreement with electrocardiography (ECG) recordings from the same mice, thus verifying that cardiac pulsation was reflected to NIRS signal as expected (Fig. 3B).

**Fig. 4:**
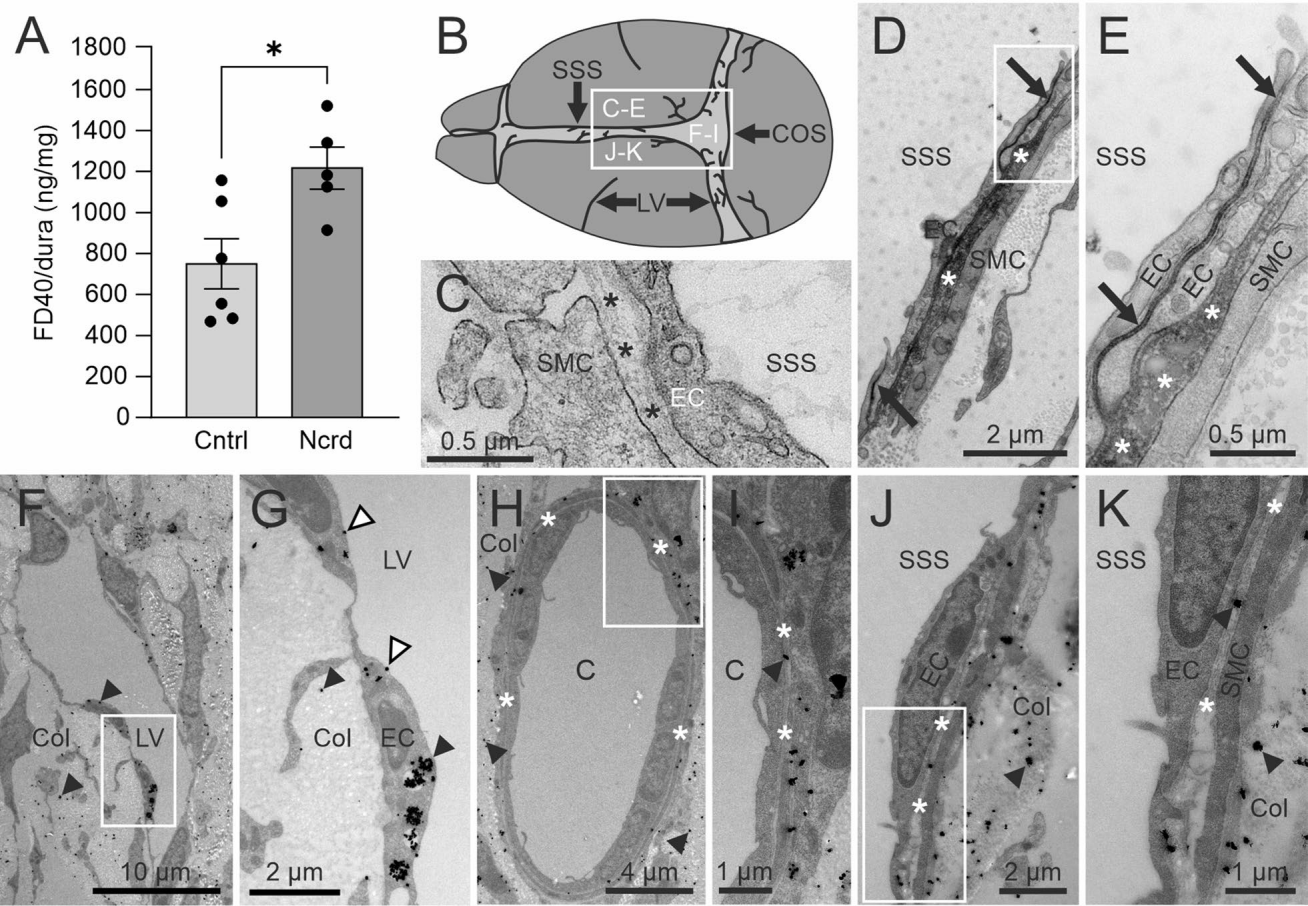
Quantification of FD40 from dorsal dura and electron microscopical imaging of meningeal efflux pathways. **A**, FD40 fluorescence measurement from homogenized dorsal dura after BPL. Unpaired t-test, *P < 0.05, n = 5–6 mice per group, filled circles represent individual mice. **B**, Schematic representation of brain cortex and dorsal dura indicating the regions in dura which were selected for EM analysis. White boxes indicate magnified regions. The superior sagittal sinus (SSS), the confluence of sinuses (COS). **C** A dural sample without HRP (negative control) displays no staining. Endothelial cells (EC), smooth muscle cells (SMC), the vascular basement membrane (asterisks). **D**, The reaction product of HRP, polymerized 3′3-diaminobenzidine (DAB), is shown as a black precipitate around the SSS. HRP is localized in the basement membrane (asterisks) and in the junctions (arrows) of ECs lining the SSS and SMCs. **E**, Higher magnification of an area in **D**, showing distribution of HRP in a junction of adjacent ECs. Similar observations from 5 different mice. **F**, Silver-enhanced nanogold-conjugated IgG (arrowheads) is localized in the region around the COS in **B**, in the dural tissue consisting of collagen (Col) fibers, and inside ECs of a meningeal lymphatic vessel (LV). **G**, Higher magnification of an area in F showing clusters of nanogold-conjugated IgG in lymphatic ECs and on the surface of the vessel lumen (white arrowheads). **H**, A blood capillary (C) in the region around the COS. **I**, Higher magnification of an area in H showing nanogold-conjugated IgG in the basement membrane of ECs. **J**, Nanogold-conjugated IgG is distributed in the region close to the SSS. **K**, Higher magnification of an area in J shows nanogold-conjugated IgG in the basement membrane between ECs and SMCs of the SSS. Similar observations from 8 different mice

Next, we investigated whether BPL affects macromolecular infiltration into the dorsal dura (Fig. 4), which has previously been shown to accumulate CSF resident molecules and to be involved in ICP regulation [40, 41]. After ~ 40 min from the CM injection, FD40 accumulated into dorsal dural tissue with significantly higher amounts (750 ± 344 ng mg^−1^ vs 1217 ± 303 ng mg^−1^) in BPL-treated mice when compared to controls receiving only saline (Fig. 4A). To further elucidate the distribution of macromolecules in relation to potential exit pathways from the dura, i.e., dural sinuses and lymphatic vessels, we performed electron microscopy from superior sagittal sinus (SSS) and confluence of sinuses (COS) areas. Horseradish peroxidase (HRP, 40 kDa) catalyzed electron dense osmophilic 3′3-diaminobenzidine (DAB) polymer was observed in the basement membranes of venous sinuses and capillaries (Figs. 4D and E, Additional file 2: Fig. S2A, B). Intense staining was also seen in endothelial junctions as well as in intracellular vesicles of endothelial cells and bordering smooth muscle cells. The staining was also present in between collagen fibrils of dural tissue (Additional file 2: Fig. S2D). In contrast, significantly larger (~ 160 kDa) nanogold-conjugated IgG molecules were only rarely seen to cross the basement membranes of blood vessels while accumulation was evident in the endothelial cells of lymphatic vessels near the COS (Fig. 4F–K, Additional file 2: Fig. S2E).

### Blood pressure lowering increases tracer efflux into deep cervical lymph nodes but reduces perivascular influx in the brain

**Fig. 5:**
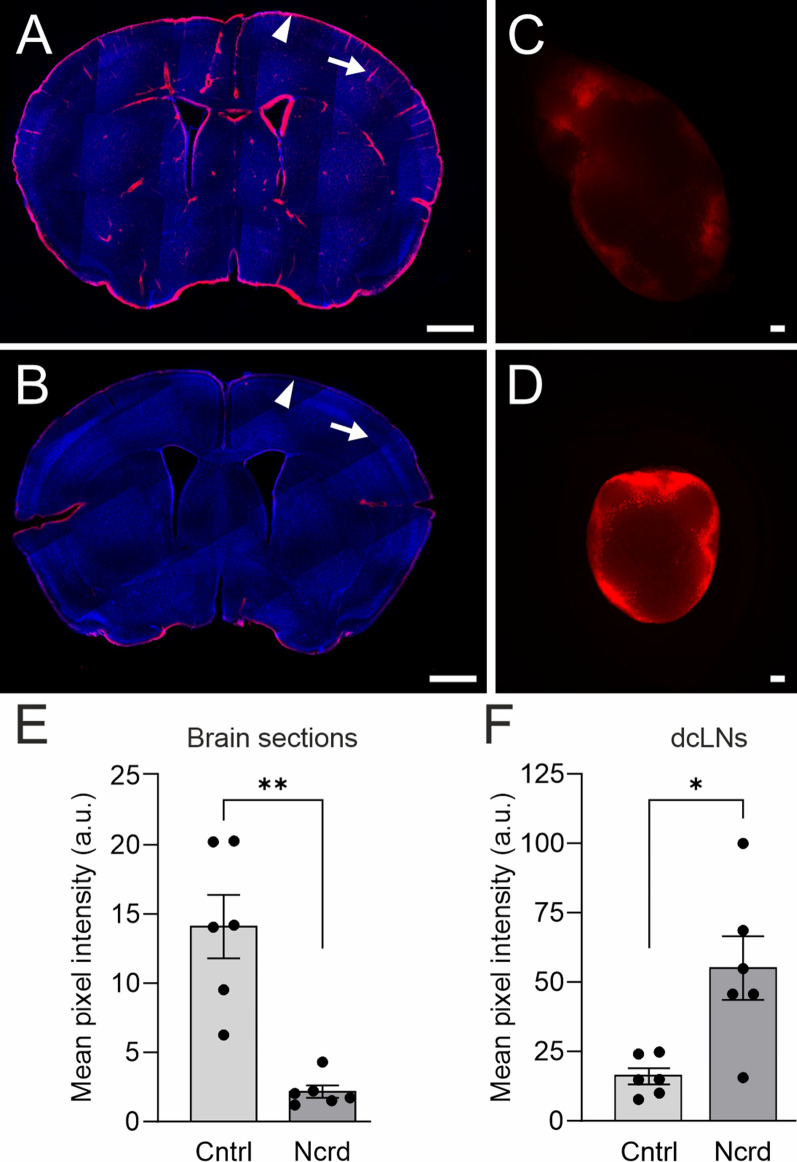
Perivascular brain influx and drainage of Texas Red-conjugated ovalbumin into dcLNs. **A**, A brain section from a control mouse showing deep parenchymal penetration (arrow) and cortically located 45 kDa Texas Red-conjugated ovalbumin (arrowhead). **B**, A brain section of a mouse after BPL displays significantly reduced presence of tracer, as quantified in E. **C**, **D**, Representative images demonstrating that BPL (**D**) improves tracer uptake into to dcLNs compared to saline-treated controls (**C**). **A – D**, Blue, DAPI; Red, 45 kDa Texas Red-conjugated ovalbumin. **E**, **F**, Fluorescence intensity quantitation from serial brain sections and dcLNs, *P < 0.05, unpaired t-test with Welch’s correction and unpaired t-test, respectively. Scale bars for brain sections and dcLNs are 1 mm and 100 µm, respectively. **A**–**F**, n = 6 mice per group. Filled circles in E and F represent individual mice

Having observed that BPL increases tracer content in the dural tissue, and that the tracer was located in the meningeal lymphatic vessel structures, we next investigated if BPL increases tracer drainage into the deep cervical lymph nodes (dcLNs), the major LNs  draining the meningeal lymphatics [12–14, 42–44]. As CSF can also enter the brain tissue along the penetrating arteries, we investigated perivascular (glymphatic) influx in brain sections [2, 45]. We injected fixable Texas Red-conjugated ovalbumin with a molecular weight similar to FD40 into the CM. We found that perivascular tracer penetration was significantly decreased in the brain sections (14.1 ± 7.0 a.u vs. 3.1 ± 1.5 a.u.) and tracer content in dcLNs was significantly increased (40.9 ± 30.6 a.u. vs 115.4 ± 75.1 a.u.) in BPL-treated mice when compared to controls receiving only saline (Fig. 5).

### Blocking deep cervical lymphatic vessels attenuates tracer outflow during blood pressure lowering

**Fig. 6:**
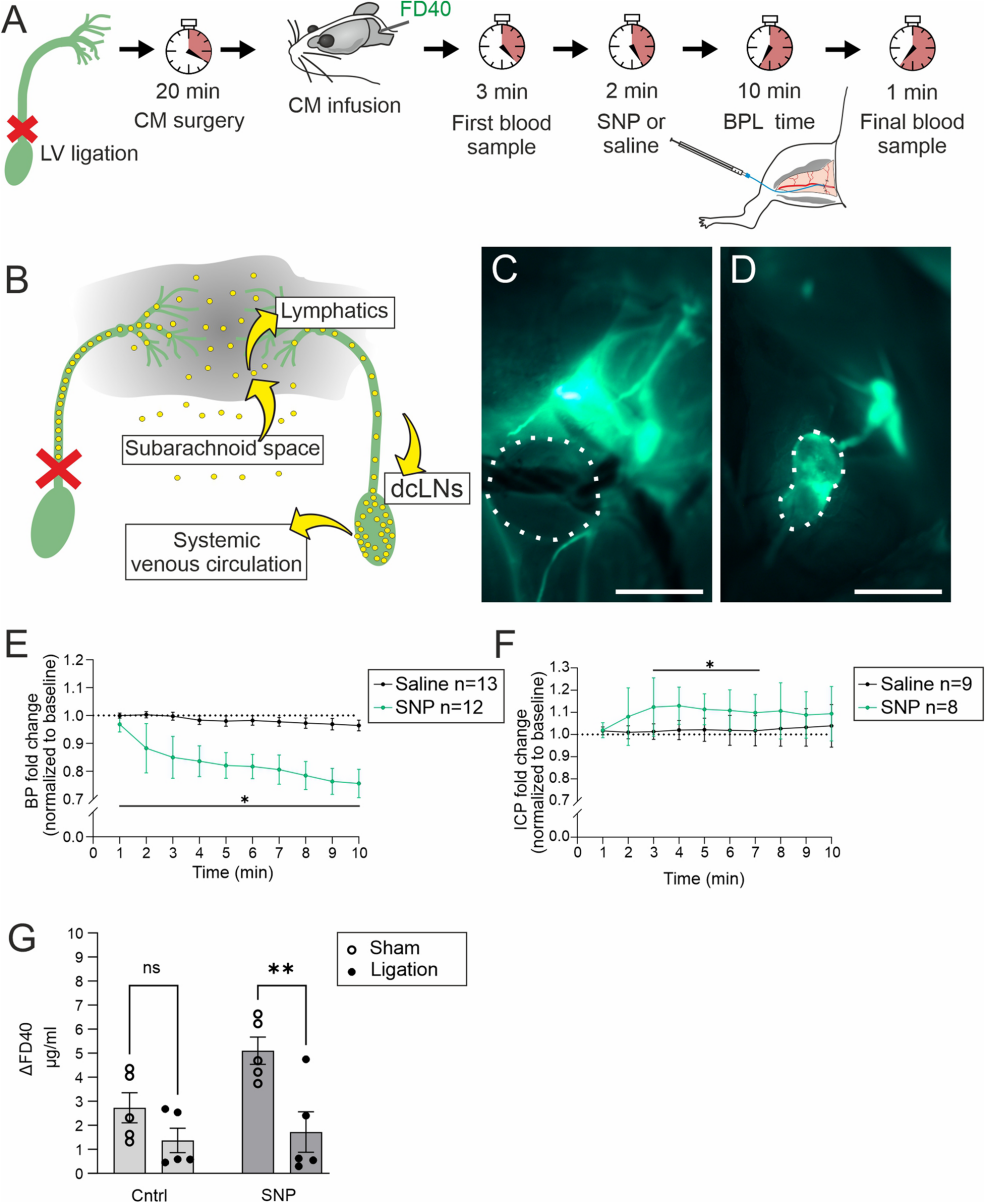
CSF tracer drainage via extracranial lymphatics to systemic blood circulation during BPL. **A**, Experimental scheme and timeline of afferent lymphatic vessel ligation, FD40 injection and blood sampling. CM infusion of FD40 began ~ 20 min after lymphatic vessel (LV) ligation, followed with first blood sampling (3 min after the infusion), 10-min BPL or saline treatment (2 min later) and final blood sampling. **B**, An illustration of afferent LV ligation (crossed red lines) which obstructs tracer uptake (yellow spheres) into a lymphatic efflux pathway (yellow arrows) and deep cervical lymph nodes (dcLNs). **C**, Drainage of FD40 into a dcLN (white dashed lines) via the afferent lymphatic vessel is blocked after ligation. **D**, FD40 drainage into a dcLN on the sham-operated side of the same animal as in **C**. **E**, **F**, Blood- (BP) and intracranial pressure (ICP) measurements during BPL with sodium nitroprusside (SNP), multiple unpaired t-tests with Welch’s correction. **G**, Concentration changes of FD40 in blood after BPL with SNP in sham-operated and ligated groups, two-way ANOVA with Šidák’s multiple comparisons. n = 5 mice per group. **C**, **D**, Scale bar, 1 mm. **E**–**G**, *P < 0.05, ns = non-significant. Filled and open circles represent individual mice (**G**)

To test the contribution of extracranial lymphatic drainage via dcLNs to the BPL effect on CNS clearance, we performed ligation of afferent lymphatic vessels (Fig. 6) that drain into dcLNs and quantified the FD40 concentration from the blood. Lymphatic efflux has been previously shown to precede blood accumulation which occurs 15–30 min after tracer injection into the CSF [46]. Therefore, we performed a 10-min BPL with sodium nitroprusside (SNP), which elicited a more immediate vasodilatory response than nicardipine (Additional file 3: Fig. S3). An i.v. injection of SNP resulted in a rapid drop in BP (max 24.0 ± 13.3%) and concomitant elevation of ICP (18.2 ± 15.0%) (Figs. 6E and F, Additional file 4: Fig. S4) while the ligation alone did not significantly elevate ICP within the estimated time window in which the separate efflux experiments were performed (Additional file 5: Fig. S5). Ligation of afferent lymphatic vessels abolished tracer drainage into dcLNs during BPL with SNP, resulting in a significant reduction of FD40 in blood (5.1 ± 1.5 µg ml^−1^ vs 1.7 ± 2.2 µg ml^−1^), thus suggesting that BPL-induced improvement of tracer absorption from the CSF is primarily through the lymphatic vasculature (Fig. 6G).

## Discussion

The major finding in this study is that acute BPL increases tracer efflux from the CSF to the lymphatic vessels that drain into the venous system through the collecting ducts. CSF clearance is highly relevant for brain homeostasis, for the recently proposed brain clearance mechanisms and in the pathogenesis of neurodegenerative diseases. However, there are no current treatment options to augment the clearance function. Our study presents novel method to enhance cerebral CSF flow in mice, suggesting that cerebral macromolecular clearance can be increased by a clinically feasible treatment.

As stated by the Monro-Kellie doctrine, a change in the volume of one compartment in the cranium must follow a compensatory volume displacement in others [39]. Vasodilation can increase vascular compliance while decreasing the compliance of the intracranial space, suggesting that even small changes in blood volume can affect ICP [47]. Accordingly, BPL resulted in an increase in ICP, a common side effect reported also in clinical studies [48–51]. Increased ICP coincides with the CSF volume displacement into blood as seen as increased tracer efflux. The pathological and sustained rise in ICP is commonly observed in traumatic brain injury [52]. However, in contrast to an acute (~ 15 min with nicardipine, ~ 4 min with SNP) and a rather mild (14 – 18%) elevation in ICP as we observed, a long-lasting (> 2 h) with more than two-fold elevation of ICP may compromise CSF drainage via the meningeal lymphatics [53, 54].

In the present study, the finding that FD40 accumulates in the dorsal dura mater upon BPL is consistent with the recent findings on the interplay of CSF and the dura, where CSF-derived antigens and substances locate in the dural parenchyma. Interestingly, the tracers are preferentially located along venous sinuses both in mice and humans [40, 55]. Given the close anatomical association of meningeal lymphatic vessels with dural sinuses [12, 56, 57], these regions are likely functional absorption sites of CSF. In support for this, disruption of lymphatic vessels from dorsal dura has been shown to play a crucial role in macromolecular clearance and cell trafficking to cervical lymph nodes [25, 58]. Our electron microscopic observations demonstrate that tracers (nanogold-conjugated IgG and HRP) are distributed in the dorsal dura mater, where they are taken up by both blood- and lymphatic endothelial cells. The basement membrane, which is continuous in blood vessels [59], but not in lymphatic capillaries, functions as a size-selective molecular sieve allowing lower molecular weight HRP molecules (40 kDa) to permeate and pass through whereas the permeation of higher molecular weight IgG molecules (160 kDa) is restricted. The finding, which is consistent with previous observations on glomerular basement membrane [60] imply that due to the relatively large surface area of dural sinuses and blood capillaries, they could have a greater contribution as an efflux pathway for smaller-sized substances. Of note, electron microscopy studies demonstrate only subcellular locations to which the tracers accumulate in the dural tissue. It has limitations in assessing tracer quantity in blood and lymphatic circulation due to very small sample volume coverage (section thickness 50–80 nm). Indeed, lymphatic drainage appears to be the main exit pathway also for smaller sized substances. Accordingly, the accumulation of Texas Red-conjugated ovalbumin (45 kDa) into dcLNs was evident in ~ 40 min following injection at CM and was significantly facilitated by BPL treatment. Furthermore, ligation of afferent lymphatic vessels draining to dcLNs significantly reduced the blood uptake of FD40 within ~ 18 min following injection into CM. In a previous study, tracers injected into the CSF exhibited a 25 to 30 min time delay before appearing in the bloodstream, whereas they were present in draining lymph nodes within 15 min [46]. Consistent with this finding, mice lacking a meningeal lymphatic system showed delayed clearance of extracellular tau-protein into blood [61]. A direct lymph transport is thus likely the main route for CSF absorption preceding a significant transit to the blood. Blood vessels may take up molecules from CSF parallelly with lymphatic drainage, but act slower and less efficiently.

Increased tracer efflux from the CSF coincided with reduced tracer inflow into the brain. This could be due to reduced amount of available tracer for paravascular entry along penetrating arteries as shown previously in awake mice [62]. It is also possible that vasodilation of pial- and penetrating arteries reduces the perivascular space, diminishing directional fluid flow. It was indeed shown recently that vasodilation by whisker stimulation reduces periarterial space and CSF flow velocity, but whisker stimulations and/or optogenetically induced vasoconstrictions in sequence lead to cumulative fluid inflow volume [63]. Thus, constantly altered vascular tone rather than chronic change (as in the present study) might be prerequisite for increased perivascular inflow as also discussed in the aforementioned article by Holstein-Rønsbo *et al*. (2023). With vasoactive agents, variances in dose–response relationship may contribute to the net direction of perivascular flow. For example, dexmedetomidine, a selective alpha 2-adrenoceptor agonist, reduces systemic PB, cerebral blood flow and ICP (in laparoscopic surgery) while facilitating brain delivery of intrathecally administered drugs [64–66]. However, dexmedetomidine has also dose-dependent vasoconstrictive effects in cerebral arterioles [67] which may explain improved CSF inflow apart from the systemic BPL-effect. In support of this, stroke associated arterial vasoconstriction enlarges the perivascular space and leads to increased CSF inflow into the brain [68]. Intracranial vasoconstriction has also been reported for nicardipine treatments in some studies [69], while in others nicardipine either increases or leaves cerebral blood flow unaltered and causes vasodilation [70–72]. For example, an i.v. dosage of 1 to 30 µg kg^−1^ increases cerebral blood flow and ICP in monkeys [71]. In the present study with 0.5 µg kg^−1^ min^−1^ nicardipine administration, we did not observe a significant alternation in cerebral oxygenation, suggesting that cerebral blood flow was not reduced. Furthermore, the observed increase in ICP likely implies a volume increase in the cerebral vasculature similar to a previous study [71]. It is thus likely that reduced perivascular influx is caused by BPL-induced cerebral vasodilation disrupting the glymphatic intake and mixing of CSF and interstitial fluid and the proposed convective flow through the interstitium [73]. As a limitation, our experiments were performed with anesthetized mice. Whether the findings also apply the non-anesthetized state warrant further studies.

## Conclusion

In conclusion, our data show that CSF dynamics can be manipulated by BPL treatment, which provides empirical justification for the Monro-Kellie doctrine in a non-pathological context. Moderate increase in ICP by BPL resulted in enhanced filtration of tracers into the bloodstream primarily through extracranial lymphatics. The data confirms that meningeal and extracranial lymphatics are essentially involved in the clearance of macromolecules from the CSF. Further investigations addressing the translational and therapeutic potential regarding the temporary enhancement of CSF absorption and lymphatic macromolecular drainage with BPL are warranted.

## Methods and materials

### Animals and housing

2- to 3-month-old C57BL/6N (Charles River) female mice were used in all experiments. All animals were housed in temperature- and humidity-controlled rooms with a 12 h light/12 h dark cycle. Teklad Global Rodent diet (Harlan Teklad, USA) and untreated tap water were offered *ad libitum*. Mice were anaesthetized with subcutaneous injection of ketamine (75 mg kg^−1^ or 100 mg kg^−1^) and xylazine (10 mg kg^−1^) (KX). Lidocaine (10 mg kg^−1^) was administered subcutaneously to surgical sites 5 min before operations.

### Cisterna magna cannulation and tracer infusion

After presurgical preparations the mice were connected to a stereotaxic device. After checking surgical site pain reactivity with forceps, neck muscles were gently separated to expose the CM. A dental needle (30G) connected to a polyethylene tube (BTPE-10) filled with saline was used to cannulate the CM as described previously [74]. FITC-dextran 40 kDa (FD40) (Sigma, FD40S-250MG; 25 mg ml^−1^), horseradish peroxidase (HRP) (77332- Sigma), nanogold-conjugated IgG (160 kDa, Aurion, 100.233, Mouse-anti-FITC Ultra Small) or 45 kDa Texas Red-conjugated ovalbumin (Thermo Fisher Scientific, O23021; 1 mg ml^−1^) were diluted in saline and withdrawn into a connecting line. Unlike FD40, Texas Red-conjugated ovalbumin could be fixed in tissues, allowing ex vivo quantitation, whereas HRP and nanogold-conjugated IgG are suitable for electron microscopical applications. 4 µl of tracers were injected with a rate of 2 µl min^−1^ into the CM by a Hamilton syringe with a 30-gauge needle filled with purified water and operated with a microinjector (KD Scientific model LEGATONANO). After infusion, excess cannula was sealed by using an electrocauter (Bovie, change-a-tip DEL1). An infrared lamp or a heat pad (set to + 37 °C) was used to help maintain the body temperature. The first blood sample was collected within ~ 5 min after the infusion, and the final sampling ~ 18 min (dcLN ligation experiments) or ~ 45 min later in the other experiments. Blood sampling was from the femoral vein (~ 160 µl for the first  sample) and carotid artery (~ 180 µl-200 µl for the second  sample) and mice were sacrificed immediately after collecting the second blood sample.

### Blood and intracranial pressure measurements

A BTPE-50 polyvinyl cannula (filled with 0.9% NaCl supplemented with 500 IU ml^−1^ heparin) was inserted into the permanently ligated left external carotid artery to access the common carotid artery as previously described [75]. For ICP, CM was cannulated with saline-containing tube, which was connected to an in-line pressure sensor (BP-102). BP and ICP were measured by using the same type of sensor. Data were collected with an iworx® IX-RA-834 (Iworx Systems Inc.) data acquisition system coupled with an iWire-BIO4 iworx® 4-Channel Biopotential Recording Module and Labscribe 4 software.

### Blood pressure lowering

Either nicardipine hydrochloride (Sigma, N7510-1G) or sodium nitroprusside (SNP) (Sigma, PHR1423-1G) was infused into the femoral vein at rates of 5 or 10 µl min^−1^, respectively. Dosages were 5.0 µg kg^−1^ min^−1^ for SNP and 0.5 µg kg^−1^ min^−1^ for nicardipine. Control groups received saline i.v. at similar rates. The i.v. infusions were 10 min for SNP and 15 min or 40 min for nicardipine.

### Deep cervical lymph node ligation experiments

DcLNs were accessed through neck incision. Afferent lymphatic vessels draining into dcLNs were ligated with surgical sutures. Sham animals received otherwise similar surgery without the ligation.

### Near infrared spectroscopy measurements

Monitoring of mouse well-being during BPL with nicardipine was performed by a functional near-infrared spectroscopy (fNIRS) device (custom-made) which can be used to monitor cerebral blood oxygenation and heart rate. It uses three laser diodes at wavelengths of 685 nm, 785 nm and 830 nm and a photodiode (Hamamatsu S5971) as a detector. The working principle of the NIRS device is based on the frequency-coding continuous wave technique, where each wavelength is labeled for a specific modulation frequency and simultaneously illuminated and then separated in the receiver [76]. The raw digitized signals were initially filtered and then fed to a digitally implemented lock-in detection and demodulation. The light source and the detector fiber probes were placed in the ear canals so that the distance between source and detector fiber tips was approximately 1 cm, and the fiber tips were positioned towards the nose. Wavelengths of 685 nm and 830 nm were used for calculating deoxygenated hemoglobin (HbR) and oxygenated hemoglobin (HbO) based on the modified Beer-Lambert law. For the heartbeat detection 785 nm was used because this wavelength gave the best cardiac pulsation among the others. We applied a band-pass filter with a cut-off frequency of 1 – 10 Hz to remove any unnecessary spikes from the pulsating signal. However, this band pass filter could not remove the baseline wandering from the signal completely; thus, it was estimated using the lower envelope. The baseline wandering was removed by subtracting the lower envelope from the signal. Next, the heart rate and heart rate variability were calculated. As the results could still contain spikes, a moving average filter (N = 100) was applied to smoothen them. To get a continuous heart rate signal over the whole measurements, the missing points were estimated using cubic spline interpolation. NIRS heartbeat detection was verified against simultaneous ECG measurements from the same mice (iWorx, IWIRE-BIO4) with subdermal platinum needle electrodes.

### Blood and dorsal dura mater collection for fluorescence measurements

160–200 µl of blood was collected and centrifuged at 1100 g for 10 min and the supernatant (serum) stored at -70 °C. Dura was cleared from blood by transcardial perfusion via the left ventricle using an infusion pump (World Precision Instruments, model NE-4000 Multi-Phaser) and a syringe filled with ice-cold PBS with heparin (500 IU ml^−1^) at 5 ml min^−1^ for 3 min. Dorsal dura, including superior sagittal and transverse sinuses, was carefully peeled off, flash frozen in liquid nitrogen and stored at -70 °C.

### Fluorescence analysis of serum and dorsal dura

Thawed serum samples were diluted with PBS 1:4. Dura samples were weighted and homogenized with a homogenizer using a metal beads (Qiagen, Tissue Lyzer LT) and centrifuged at 12 400 g for 1 h at RT. The supernatant was diluted with PBS and assayed in triplicates using a fluorometer (PerkinElmer, VICTOR3V 1420 Multilabel counter) with excitation/emission of 485 nm / 535 nm (25 nm band-pass filter). Serum and dura without FD40 served as negative control.

### Microscopical fluorescence analysis from brain and deep cervical lymph nodes

Brain and dcLNs containing fixable 45 kDa Texas Red-conjugated ovalbumin were isolated and immersion-fixed in 4% paraformaldehyde in PBS overnight at + 4 °C. On the following day, samples were transferred to PBS with 0.02% sodium azide until further processing. Brains were sectioned coronally from bregma + 1.8 mm to –2.4 mm into 100-µm sections using a vibratome (VT1200S, Leica) and collected in PBS (with 0.02% sodium azide). Every 6th section was selected, and incubated in PBS containing 4′,6-diamidino-2-phenylindole (DAPI, dilution 1:1000) overnight at + 4 °C on a shaker. Brain slices were mounted on glass slides, covered with a cover slip, and stored at + 4 °C until imaging. Brain and lymph nodes containing ovalbumin-Texas Red were imaged using an AxioZoom V16 stereomicroscope (Carl Zeiss) equipped with an Orca-Fusion BT sCMOS camera (Hamamatsu Systems) using Zen 3.3 pro software (Carl Zeiss). The objective used was Plan Neofluar Z 2.3 × and Plan Neofluar Z 1.0x, respectively and imaging parameters were the same for all the samples. Quantitative analysis of the acquired images was performed using the Fiji processing package of Image J2 software (Version 1.53, National Institute of Health). For perivascular influx analysis, the background was subtracted uniformly from the brain sections. The slices were manually outlined, mean pixel intensity was measured and the average value of 7 sections per animal was reported. A free hand -tool was used to make a region of interest and mean pixel intensities of dNLs from each animal were measured and the average value was reported.

### Tissue processing for transmission electron microscopy

Mice were perfused with ice-cold PBS to remove blood and then fixed with ice-cold 1% glutaraldehyde, 4% paraformaldehyde in 0.1 M phosphate buffer via transcardial perfusion followed by collection of dorsal dura. HRP samples were then washed with PBS before incubation with fresh 0.05% 3′3-diaminobenzidine PBS solution (DAB, Sigma, D5637). After 20 min at RT DAB-solution was aspirated and the samples were immersed in 0.05% DAB-peroxidase-PBS solution for 30 min at RT. The staining reaction was terminated by washing the samples with ice-cold PBS and post-fixed with ice-cold fixative. HRP samples were post-fixed with 1% osmium tetroxide (OsO_4_) and 1.5% tetrapotassium hexacyanoferrate (C_6_FeK_4_N_6_) 2 h at + 4 °C before dehydration and LX112 resin infiltration. Post-staining was done with trilead tricitrate (C_12_H_10_O_14_Pb_3_). Samples without HRP were treated as above but were devoid of DAB treatment, including 1% uranyl acetate (C_4_H_6_O_6_U) staining. Fixated nanogold-conjugated IgG samples were washed with distilled water for four times for 10 min, followed by osmication (0.3%, 1 h) and washed with distilled water three times for 10 min. Samples were then processed as per the instructions given in the AURION R-Gent SE-EM package; 20 drops of the enhancer solution were dripped into a 1.5 ml test tube. 1 drop of the developer solution was then added by dripping. Samples were floated in the enhancement mixture on a rocking table for 2 h at RT and then washed extensively with distilled water for at least three times for 10 min and processed further with dehydration and LX112 resin infiltration. Transmission electron microscopy imaging was performed at the EM Core Facility at Biocenter Oulu, Finland with Tecnai Spirit microscope (Fei Europe) equipped with a Quemesa CCD camera (Olympus Soft Imaging Solutions GMBH).

### Statistical analyses

Values are mean ± standard error of mean or 95% confidence interval (data shown across multiple timepoints). Unpaired t-test was used for comparison of two groups. In the case of unequal variances Welch’s correction was used. If data was not normally distributed as shown by Shapiro–Wilk test, Mann–Whitney *U* test was used. In experiments with more than two groups, ordinary two-way analysis of variance (ANOVA) followed by post hoc Šidák’s multiple comparisons testing was employed. For multiple timepoints, where each timepoint was compared between two groups, multiple unpaired t-tests with Welch’s correction were used. Alternatively, ordinary one-way ANOVA with repeated measures and Greenhouse–Geisser correction followed by Dunnett’s multiple comparisons test was employed, where each timepoint was tested against the first timepoint after the baseline. Values for multiple timepoints are presented as fold changes instead of absolute values due to high physiological variability of baseline values in individual mice. *P* < 0.05 was considered statistically significant. All analyses were performed with GraphPad Prism (version 9.3.1, GraphPad Software, Inc.).

### Supplementary Information

**Additional file 1: Figure S1.** Absolute values of blood- (BP) and intracranial pressures (ICP) of mice treated with nicardipine (**A**, **B**, Ncrd) or saline (**C**, **D**, Cntrl) infusion. Each curve represents measurement from a single mouse sampled at one minute interval. n = 9 (**A**), n = 5 (**B**), n = 4 (**C**) and n = 6 mice (**D**). Note decline in BP (**A**) and increase in ICP (**B**) absolute values in individual mice after Ncrd infusion (dashed line). Nicardipine infusion increases heart rate as shown in Fig. 3B.

**Additional file 2: Figure S2.** Distribution of CSF tracers, HRP and nanogold-conjugated IgG, in the dorsal dura mater. **A**, 40 min after injection into the CM, HRP (black arrow) distributes into the basement membrane of blood capillaries (C). White frame indicates a magnified region in **B**. **B**, HRP is taken up by vesicles (black arrowheads) of endothelial cells (ECs) bordering a capillary. **C**, HRP in the junction (white arrowheads) of ECs bordering the superior sagittal sinus (SSS). The tracer is also taken up into junctional vesicles (black arrowhead). **D**, HRP distributes within collagen (Col) fibers in dural tissue (arrows) and endothelial basement membrane (asterisks). **E**, Nanogold-conjugated IgG molecules inside an EC (black arrowheads), in the lumen of the SSS (white arrowhead), in the blood endothelial basement membrane (open arrowhead) and in the fibrillar collagen (Col) matrix (arrows).

**Additional file 3: Figure S3.** 10-min blood pressure (BP) measurement during an i.v. infusion of nicardipine (Ncrd) or sodium nitroprusside (SNP). Flow rate was 10 µl min^-1^. One-way ANOVA with repeated measures and Dunnett’s multiple comparisons. *P < 0.05, ns = non-significant.

**Additional file 4: Figure S4.** Absolute values of blood- (BP) and intracranial pressures (ICP) and heart rate of mice treated with sodium nitroprusside (SNP; in panels **A**, **B**, **E**) or saline infusion (Cntrl; **C**, **D**, **F**). Each curve represents measurement from a single mouse sampled at minute interval. n = 12 (**A**), n = 8 (**B**), n = 13 (**C**), n = 9 (**D**), n = 8 (**E**) and n = 9 mice (**F**). Note decline in BP (**A**) and increase in ICP (**B**) absolute values in individual mice after SNP infusion (dashed line).

**Additional file 5: Figure S5.** ICP after afferent lymphatic vessel ligation. ICP measurement was done in every minute after completion of ligation surgery (red arrow). ICP data (black filled circles) was normalized to the ICP value before the lymphatic vessel ligation (baseline). Note that the ligation of afferent lymphatic vessel does not increase the ICP above the baseline value.

## Data Availability

The datasets used and/or analyzed in this study are available from the corresponding author on reasonable request.

## Abbreviations

AD: Alzheimer’s disease
ANOVA: Analysis of variance
BP: Blood pressure
BPL: Blood pressure lowering
CM: Cisterna magna
CNS: Central nervous system
COS: Confluence of sinuses
CSF: Cerebrospinal fluid
DAB: 3′3-Diaminobenzidine
DAPI: 4′,6-Diamidino-2-phenylindole
dcLN: Deep cervical lymph node
ECG: Electrocardiography
EC: Endothelial cell
FD40: FITC-dextran 40 kDa
HbO: Oxygenated hemoglobin
HbR: Deoxygenated hemoglobin
HRP: Horseradish peroxidase
ICP: Intracranial pressure
i.v.: Intravenous
KX: Ketamine-xylazine
NIRS: Near-infrared spectroscopy
SMC: Smooth muscle cell
SNP: Sodium nitroprusside
SSS: Superior sagittal sinus
