# Supplementary figures and images for: Blood pressure lowering enhances cerebrospinal fluid efflux to the systemic circulation primarily via the lymphatic vasculature
Source: Fluids Barriers CNS. 2024 Jan 26;21:12. doi: 10.1186/s12987-024-00509-9 (PMC10821255; doi:10.1186/s12987-024-00509-9)

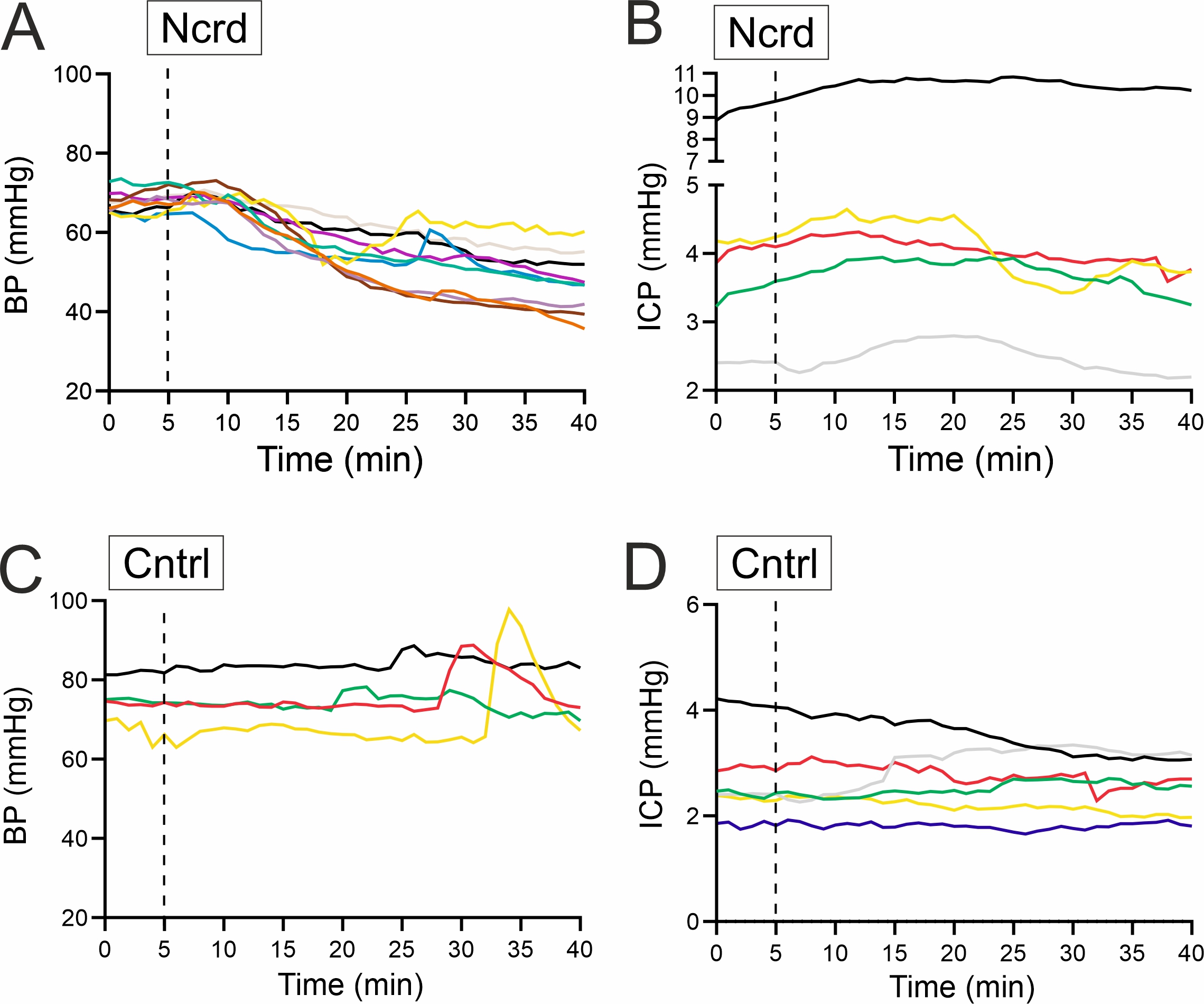

Supplement: Supplementary file 1 — Additional file 1: Figure S1. Absolute values of blood- (BP) and intracranial pressures (ICP) of mice treated with nicardipine (A, B, Ncrd) or saline (C, D, Cntrl) infusion. Each curve represents measurement from a single mouse sampled at one minute interval. n = 9 (A), n = 5 (B), n = 4 (C) and n = 6 mice (D). Note decline in BP (A) and increase in ICP (B) absolute values in individual mice after Ncrd infusion (dashed line). Nicardipine infusion increases heart rate as shown in Fig. 3B. [file 12987_2024_509_MOESM1_ESM.jpg]

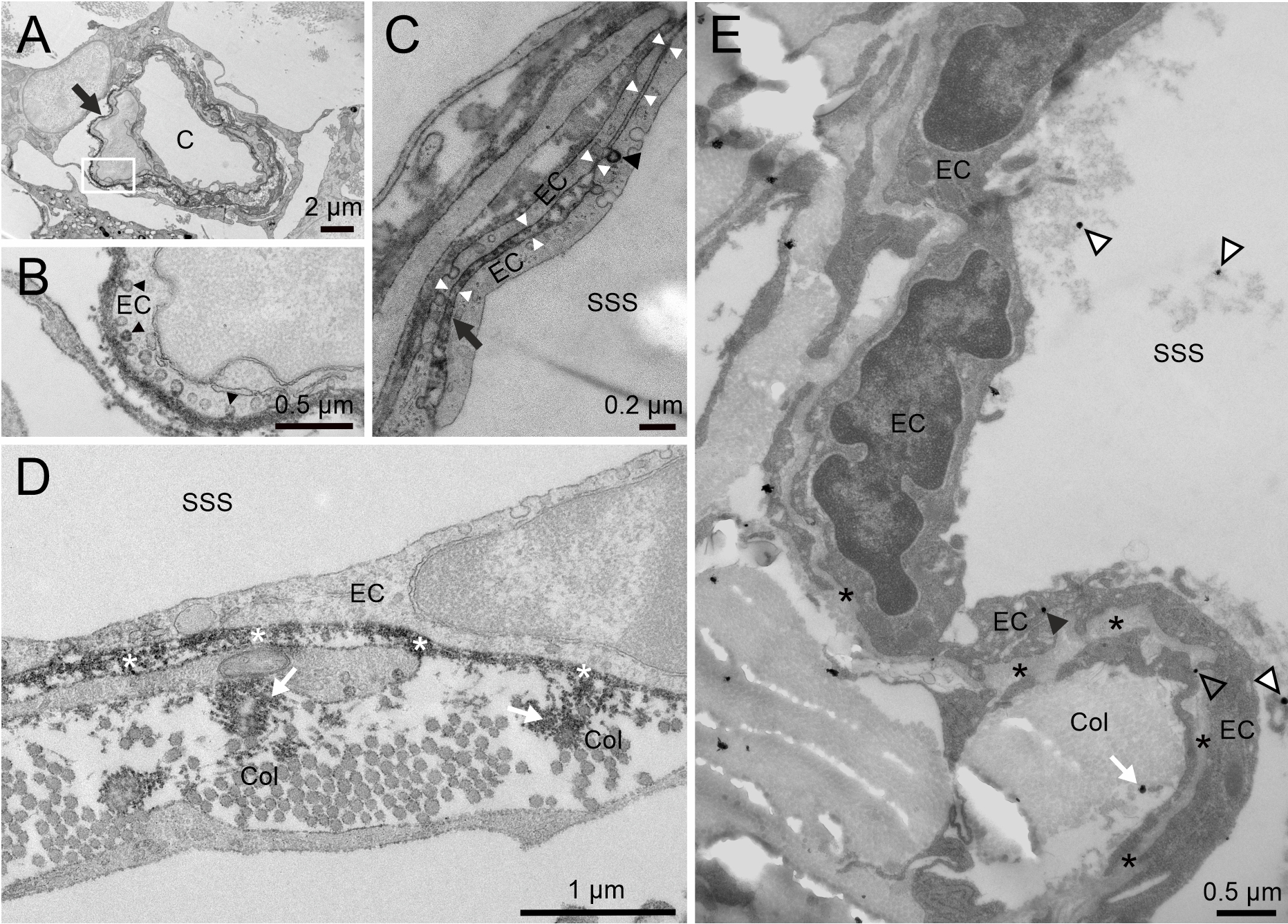

Supplement: Supplementary file 2 — Additional file 2: Figure S2. Distribution of CSF tracers, HRP and nanogold-conjugated IgG, in the dorsal dura mater. A, 40 min after injection into the CM, HRP (black arrow) distributes into the basement membrane of blood capillaries (C). White frame indicates a magnified region in B. B, HRP is taken up by vesicles (black arrowheads) of endothelial cells (ECs) bordering a capillary. C, HRP in the junction (white arrowheads) of ECs bordering the superior sagittal sinus (SSS). The tracer is also taken up into junctional vesicles (black arrowhead). D, HRP distributes within collagen (Col) fibers in dural tissue (arrows) and endothelial basement membrane (asterisks). E, Nanogold-conjugated IgG molecules inside an EC (black arrowheads), in the lumen of the SSS (white arrowhead), in the blood endothelial basement membrane (open arrowhead) and in the fibrillar collagen (Col) matrix (arrows). [file 12987_2024_509_MOESM2_ESM.jpg]

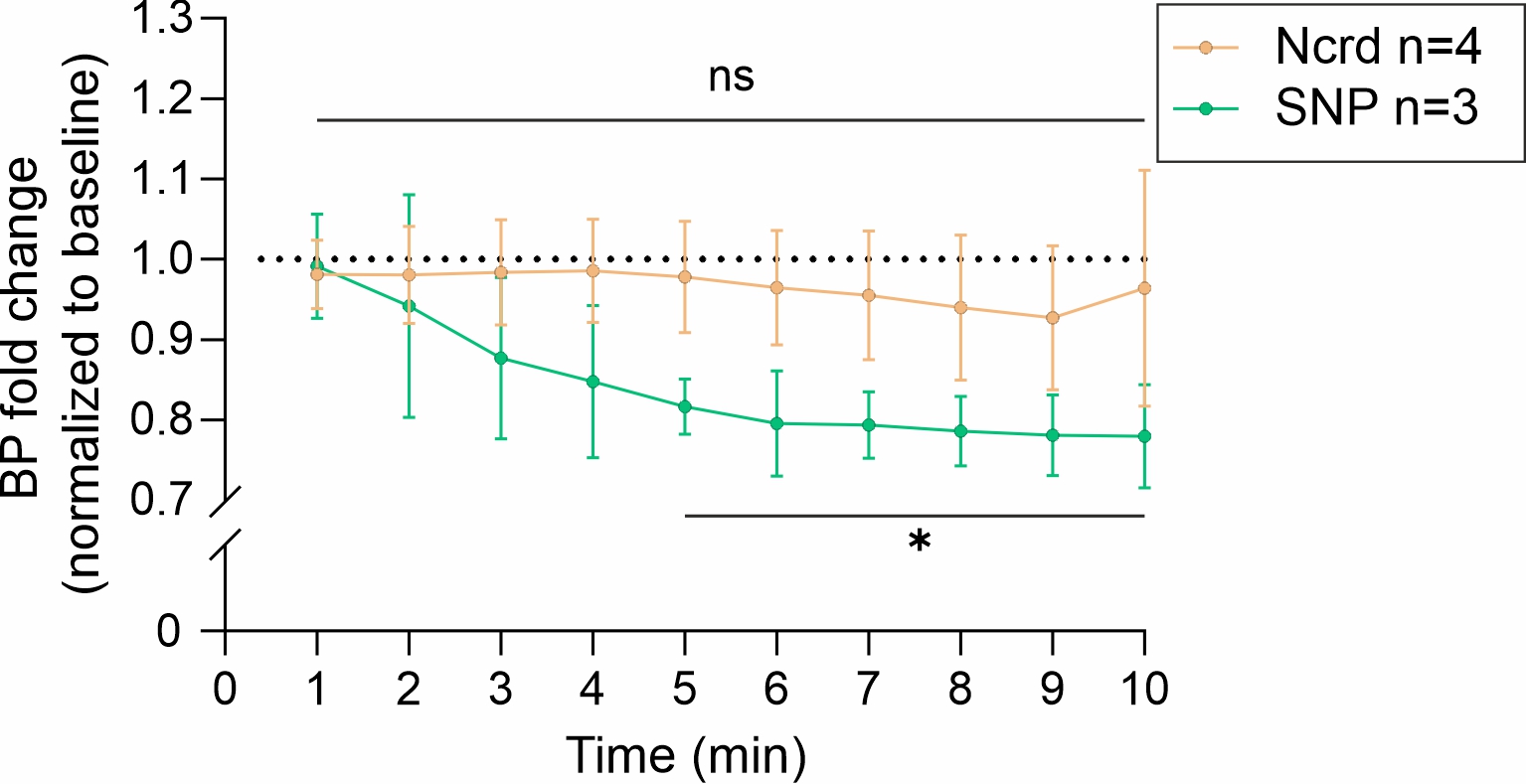

Supplement: Supplementary file 3 — Additional file 3: Figure S3. 10-min blood pressure (BP) measurement during an i.v. infusion of nicardipine (Ncrd) or sodium nitroprusside (SNP). Flow rate was 10 µl min-1. One-way ANOVA with repeated measures and Dunnett’s multiple comparisons. *P < 0.05, ns = non-significant. [file 12987_2024_509_MOESM3_ESM.jpg]

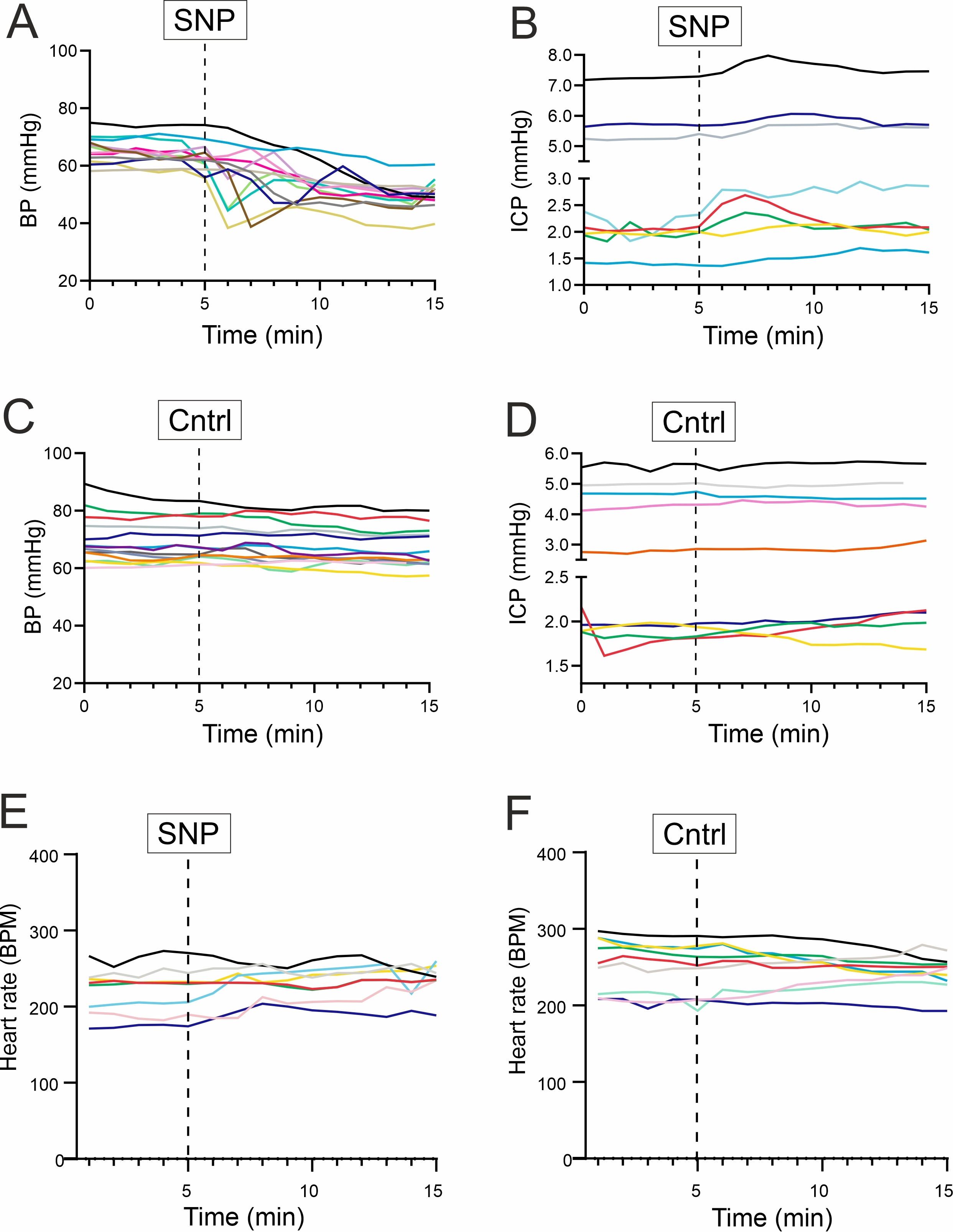

Supplement: Supplementary file 4 — Additional file 4: Figure S4. Absolute values of blood- (BP) and intracranial pressures (ICP) and heart rate of mice treated with sodium nitroprusside (SNP; in panels A, B, E) or saline infusion (Cntrl; C, D, F). Each curve represents measurement from a single mouse sampled at minute interval. n = 12 (A), n = 8 (B), n = 13 (C), n = 9 (D), n = 8 (E) and n = 9 mice (F). Note decline in BP (A) and increase in ICP (B) absolute values in individual mice after SNP infusion (dashed line). [file 12987_2024_509_MOESM4_ESM.jpg]

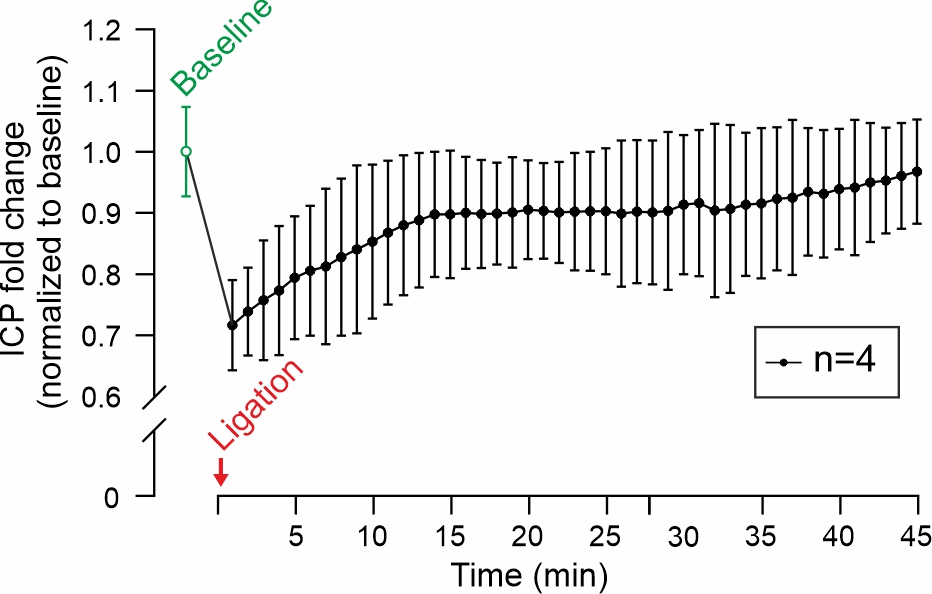

Supplement: Supplementary file 5 — Additional file 5: Figure S5. ICP after afferent lymphatic vessel ligation. ICP measurement was done in every minute after completion of ligation surgery (red arrow). ICP data (black filled circles) was normalized to the ICP value before the lymphatic vessel ligation (baseline). Note that the ligation of afferent lymphatic vessel does not increase the ICP above the baseline value. [file 12987_2024_509_MOESM5_ESM.jpg]
